# The data on the effective qualifications of teachers in medical sciences: An application of combined fuzzy AHP and fuzzy TOPSIS methods

**DOI:** 10.1016/j.dib.2018.10.165

**Published:** 2018-11-03

**Authors:** Abolfazl Khoshi, Hossein Shams Gooshki, Norouz Mahmoudi

**Affiliations:** a Associated professor, Department of Islamic Culture and Education, Faculty of Medicine, Baqiyatallah University of Medical Sciences, Tehran, Iran; b Quran and Hadith Research Center, Baqiyatallah University of Medical Sciences, Tehran, Iran; c Ph.D Student, Student Research Committee, Department of Environmental Health Engineering, School of Public Health, Iran University of Medical Sciences, Tehran, Iran; d Health Research Center, Life style Institute, Baqiyatallah University of Medical Sciences, Tehran, Iran

**Keywords:** Education, Fuzzy, Educational services, TOPSIS

## Abstract

In this data article, hybrid fuzzy Analytic Hierarchy Process (AHP) and fuzzy Technique for Order of Preference by Similarity to Ideal Solution (TOPSIS) were used to prioritize the effective qualifications of teachers of medical courses at university from the viewpoint of students of allied medicine school in Tehran University of Medical Sciences in 2013–2014. To obtain data, 200 students of allied medicine school of Tehran University of Medical Sciences were selected using random sampling method, and surveyed according to Cochran׳s formula. Data collection tools were two research-based questionnaires divided to technical, professional and individual parts. Content validity was approved by the experts. Reliability was confirmed by calculating the Cronbach׳s alpha (α=0, 85) in order to measure the degree of internal cohesion.

**Specifications Table**

**Table:** 

Subject area	Social Sciences
More specific subject area	Medical education
Type of data	Tables
How data was acquired	This data was acquired from 200 students of Tehran University of Medical Sciences.
Data format	Raw and analyzed
Experimental factors	Validity of this questionnaire was approved by interviewing with experts and professors. Its reliability was approved by Cronbach alpha (α= 0.85) for measuring the degree of internal cohesion of questionnaires.
Experimental features	The questionnaire includes 17 indexes for effective teaching in three dimensions of technical (5 indexes), professional (5 indexes) and personal qualifications (7 indexes).
Data source location	Tehran, Iran
Data accessibility	Data are included in this article
Related research article	*Z. Demirtaş, S. Arslan, A. Eskicumali, E. Civan, Teachers’ evaluations about elective mathematic applications for 5th and 6th grade curriculum, Procedia-Social and Behavioral Sciences. 174 (2015) 4074–4082**[1]*.

**Value of the data**

• There has not been a complete study about the classification of effective qualifications of teachers [1]. The data in this article provides such information.
• This data can be useful teachers of medical courses at university to enhance their ability.
• This data can be useful for medical educational programs to enhance their quality.

## 1 Data

The demographic data of scholars is shown in Table 1. Table 2 shows the data from calculating the weights of other criteria. The positive and negative ideal points were also determined regarding the equations 9 and 10 and the distance of each index from the ideal points was calculated. By considering the calculated distances and finally criterion of similarity of ranking the main indexes for the given case has been measured. The data have been shown in Table 3. Accordingly, these three main indexes have been ranked by AHP integrated algorithm and fuzzy TOPSIS and the ability to evaluate the students has been determined as the most and appearance as the least important factors.

**Table 1:** Demographic data of scholars.

**Characteristics**	**Value**
Mean of age	44 years old
Mean of work experience	15 years
Gender	221 male and 3 female
Academic rank	Professor 3, associate professor 4, assistant professor 9, instructor 8
The number of participants	24 of professors and scholars in religion courses

**Table 2:** Weights being calculated for the investigated criteria by using AHP method.

**Investigated criteria**	**Non fuzzy scale of criteria**	**Weighted vector criteria**	**Fuzzy weighted criteria**
Technical qualifications	(1.021, 1.34, 1.19)	(0.3, 0.39, 0.49)	0.3
Professional qualifications	(1.07, 1.19, 1.33)	(0.31, 0.39, 0.48)	0.38
personal qualifications	(0.66, 0.7, 0.79)	(0.28, 0.23, 0.19)	0.23

**Table 3:** The scales of distances from ideal points and the criterion of similarity calculated for main indexes.

**Indexes related to the mentioned criteria**	**The criteria related to effective qualification**	$d_{i}^{-}$	$d_{i}^{+}$	**Similarity**	**Ranking**
Scientific literacy	Technical qualifications	0.906	0.864	0.512	15
Research literacy		0.921	0.652	0.586	6
Academic level		0.866	0.721	0.546	11
Teaching experience		0.897	0.683	0.568	7
Familiar with reliable resources		0.752	0.725	0.509	16
The ability to use various and modern teaching methods	Professional qualification	0.694	0.469	0.597	5
Determining contents, organizing and collecting the materials		0.95	0.581	0.621	3
Ability to evaluate the students		0.824	0.824	0.5	1
Managing the class		0.876	0.704	0.554	9
Ability to explain the materials to students		0.932	0.493	0.654	16
Positive attitudes to the students	Personal qualification	0.873	0.522	0.626	2
Creativity in teaching		0.789	0.645	0.550	10
Observing the professional ethics		0.905	0.806	0.529	12
Ability to accept the criticism		0.823	0.752	0.523	13
Ability to make a positive communication with students		0.987	0.652	0.602	4
Flexibility		0.862	0.691	0.555	8
Appearance		0.726	0.791	0.479	17

## 2 Experimental design, materials, and methods

To obtain this data, samples were included the medical students of Tehran University of Medical Sciences. Based on De Morgan model, 200 students were determined from 420 students during 2013–2014. We explained our objectives to the students, and ensured them that their data will be used anonymously. For ranking the three indexes of technical, professional and personal qualifications in teachers of medical course from the point of view of students, six criteria were considered. The weights of six criteria were determined by AHP method and then three main indexes were ranked by using these weights and fuzzy TOPSIS. For this purpose, in the first stage, based on the decision tree shown in Table 4, a questionnaire including pair comparison was designed to determine the weights of criteria and handed out among the participants. According to Table 3, the mentioned questionnaire includes 17 indexes for effective teaching in three dimensions of technical (5 indexes), professional (5 indexes) and personal qualifications (7 indexes). Validity of this questionnaire was approved by interviewing with experts and professors and its reliability was approved by Cronbach alpha (α= 0.85) for measuring the degree of internal cohesion of questionnaires. The internal cohesion means the questions which are considered for measuring a common concept should have practically similar points.

**Table 4:** Criteria and indexes for prioritizing the effective qualifications of teacher.

**The criteria related to effective qualification**	**Indexes related to the mentioned criteria**
Technical qualifications	Scientific literacy
	Research literacy
	Academic level
	Teaching experience
	Familiar with reliable resources
Professional qualifications	The ability to use various and modern teaching methods
	Determining contents, organizing and collecting the materials
	Ability to evaluate the students
	Managing the class
	Ability to explain the materials to students
Personal qualifications	Positive attitudes to the students
	Creativity in teaching
	Observing the professional ethics
	Ability to accept the criticism
	Ability to make a positive communication with students
	Flexibility
	Appearance

After completing the questionnaires, using the formulae of MATLAB, first the rate of inconsistency of comparison matrix was calculated and the answers which their inconsistency rate was more than 0.1 were excluded. By means of geometric means formula, the matrix of individuals’ comments was integrated into one matrix. In next stage, the final matrix entered to the software and final weight for each criterion was calculated by Buckley [2] that is presented in Table 5.

**Table 5:** Weighting the criteria by using AHP technique.

**Criterion**	**Pair comparison matrix**		
	**Professional qualifications**	**Technical qualifications**	**personal qualifications**
Personal qualifications	(1.21, 1.49, 1.74)	(0.88, 1.14, 1.37)	(1,1,1)
Technical qualifications	(1.7, 1.94, 2.05)	(1,1,1)	(0.73, 0.88, 1.14)
Professional qualifications	(1,1,1)	(0.49, 0.42, 0.59)	(0.58, 0.67, 0.83)

In the next step, the fuzzy TOPSIS for ranking the main indexes was used. For this purpose, the participants were asked to assess the importance of each option by using linguistic variables presented in Table 6 and fuzzy numbers corresponding to them.

**Table 6:** Linguistic variable used to rank the options.

**Linguistic variable**	**Triangular fuzzy numbers**
Very weak	(0,1,3)
Weak	(1,3,5)
Average	(3,5,7)
Good	(5,7,9)
Very good	(7,9,10)
